# Bluetongue Virus in Wild Deer, Belgium, 2005–2008

**DOI:** 10.3201/eid1605.091217

**Published:** 2010-05

**Authors:** Annick Linden, Fabien Grégoire, Adrien Nahayo, David Hanrez, Bénédicte Mousset, Audrey Laurent Massart, Ilse De Leeuw, Elise Vandemeulebroucke, Frank Vandenbussche, Kris De Clercq

**Affiliations:** University of Liège, Liège, Belgium (A. Linden, F. Grégoire, A. Nahayo, D. Hanrez, B. Mousset, L. Massart); Veterinary and Agrochemical Research Centre, Ukkel, Belgium (I. De Leeuw, E. Vandemeulebroucke, F. Vandenbussche, K. De Clercq)

**Keywords:** Bluetongue virus, wildlife, emergence, viruses, cervids, deer, red deer, roe deer, Belgium, dispatch

## Abstract

To investigate bluetongue virus serotype 8 infection in Belgium, we conducted a virologic and serologic survey on 2,416 free-ranging cervids during 2005–2008. Infection emerged in 2006 and spread over the study area in red deer, but not in roe deer.

Bluetongue virus serotype 8 (BTV-8) spread throughout western Europe in 2006. Belgium reported its first case in farm ruminants in 2006. Because some cervid species may be seriously affected by BTV and because they may be reservoir hosts (*1*), we conducted a large-scale survey of BTV-8 in Belgium.

## The Study

Postmortem examinations were conducted on 1,620 red deer and 796 roe deer shot by hunters during hunting seasons in 2005–2008 (Technical Appendix). Sex, age, body condition, and macroscopic aspects of hooves, mucosae, and internal organs were recorded. Blood and spleen samples were obtained.

Antibodies against virus protein 7 were detected by using a competition ELISA kit (ID-VET, Montpellier, France). Results were expressed as percentage negativity compared with kit negative control serum, and cutoff values were established. Serum samples from 80 red deer and 40 roe deer were also analyzed by using a virus neutralization (VN) test for BTV-1 and BTV-8. Spleen samples obtained in 2006 and 2007 were used for detection of BTV RNA segment 5 and cellular β-actin transcripts by reverse transcription–quantitative PCR (RT-qPCR) according to a modified procedure of Toussaint et al. (*2*).

To assess performance of the ELISA, we performed receiver operating characteristic analysis. To estimate effects of potential factors (sex and age, year, month and area of sampling) on risk for seropositivity, we used a multivariate logistic regression model. Between-group differences were assessed by using the χ^2^ test.

A total of 237 pairs of ELISA and RT-qPCR results from red deer were used for receiver operating characteristic analysis, which yielded an area under the curve of 0.811 and a cutoff value for the ELISA that maximized sensitivity (86%) and specificity (98%). Serologic status was defined as positive (<66%), doubtful (>66%–<75%), or negative (>75%) and was comparable to that found for domestic ruminants (*3*). For BTV-8, concordance between ELISA and VN results was 95% for red deer and 82% for roe deer. Neutralizing antibodies to BTV-1 were not detected.

From 2006 on, no gross lesions compatible with bluetongue disease were found. In 2005, all serum samples were negative. For hunting seasons in 2006, 2007, and 2008, seroprevalences were 1.51% (95% confidence interval [CI] 0.89%–2.07%), 52.33% (95% CI 49.91%–54.78%), and 33.95% (95% CI 31.64%–36.26%) for red deer and 2.56% (95% CI 1.43%–3.60%), 2.75% (95% CI 1.62%–3.90%), and 1.67% (95% CI 0.75%–2.51%) for roe deer and showed a significant difference between species (p<0.0001 by Cochran-Mantel test).

**Figure 1:**
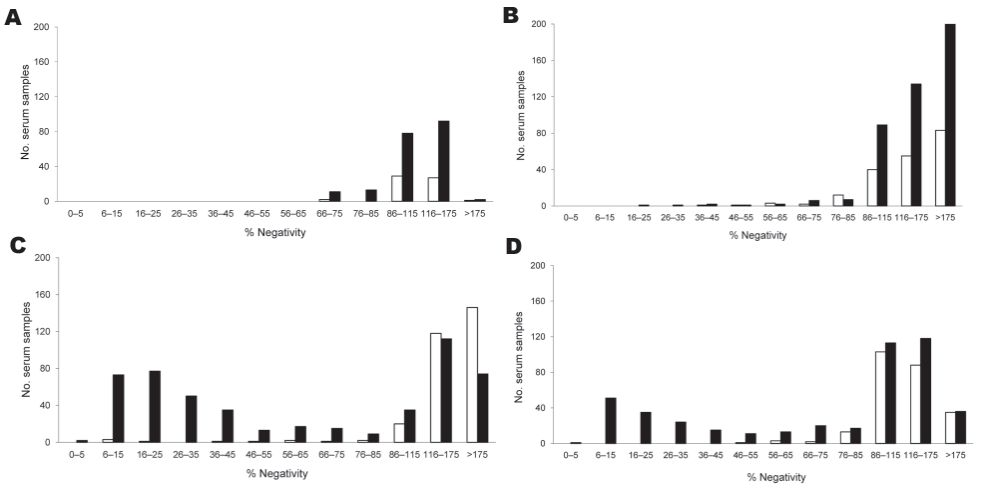
Frequency distribution of results of a competitive ELISA for detecting antibodies against bluetongue virus in serum samples from roe deer (white columns) and red deer (black columns) during the hunting seasons of A) 2005, B) 2006, C) 2007, and D) 2008, Belgium. Hunting was conducted in 30 (area 12,851 km^2^) of 37 (area 16,844 km^2^) forest districts known to contain wild cervids. The study population of wild cervids in southern Belgium (49°30′N–50°48′N) is estimated to be ≈11,000 red deer (*Cervus elaphus*) and ≈33,000 roe deer (*Capreolus* *capreolus*). Serum samples with a percentage negativity value (relative to the negative control serum) <66 were considered positive.

**Figure 2:**
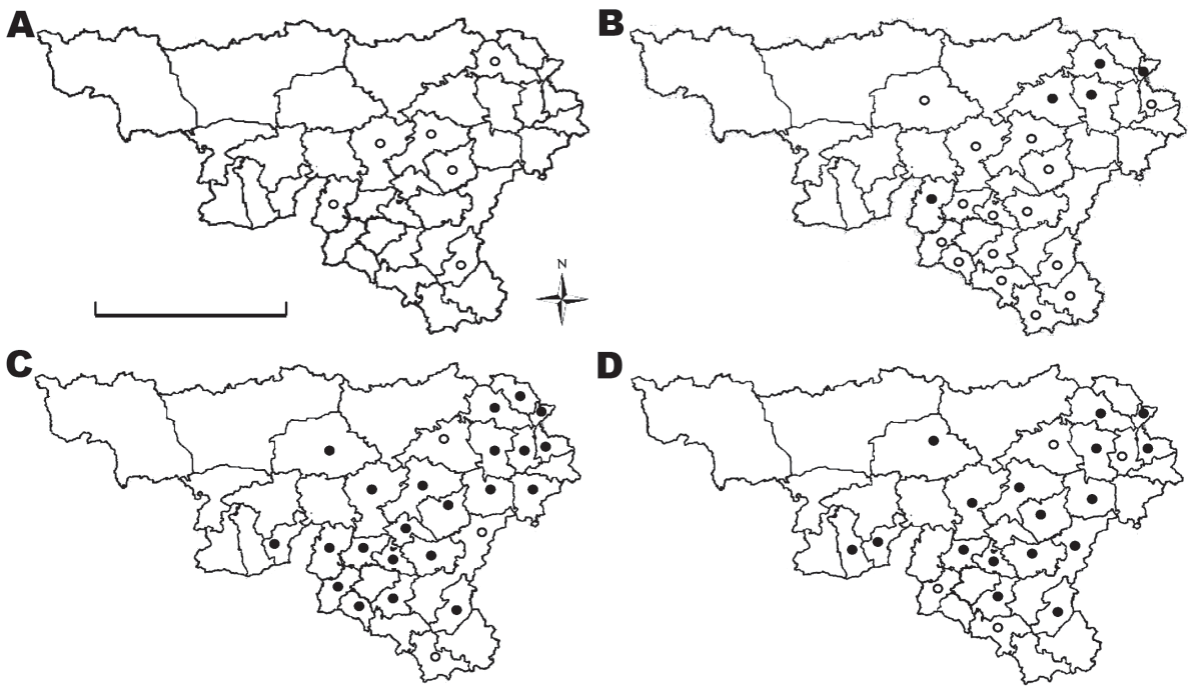
Distribution of red deer samples obtained in Belgium (Wallonia) in A) 2005, B) 2006, C) 2007, and D) 2008, and location of forest districts. White circles indicate districts where only seronegative animals were detected, and black circles indicate districts where seropositive animals were detected. Scale bar indicates 100 km.

Yearly profiles of humoral immune responses are shown in Figure 1. Unimodal negatively skewed distributions of percentage negativity in 2005 and 2006 likely reflect seronegative populations. Conversely, bimodal profiles in 2007 and 2008 are compatible with ongoing infections in the red deer population. Spatial evolution of humoral responses in red deer is shown in Figure 2. In 2006, seropositive animals were detected in only 5 districts, of which 4 were the most eastern districts among the 20 sampled; deer in most districts were infected in 2007 (22/25) and 2008 (17/21).

In red deer, multiple logistic regression analysis showed that risk for seropositivity was significantly affected by age (χ^2^ 84.53, p<0.0001), year (χ^2^ 282.75, p<0.0001), and location of sampling (χ^2^ 63.10, p<0.0001), but not by sex (χ^2^ 0.19, p>0.90) or month of sampling (χ^2^ 2.45, p>0.45). Seroprevalence was lower for juveniles than for subadults (odds ratio 2.11, 95% CI 1.47–3.04) and adults (odds ratio 3.79, 95% CI 2.85–4.62). The decrease in 2008 was significant only for juveniles, and the seropositivity gradient decreased toward southern part of the study region (Table). For roe deer, risk for seropositivity was not influenced by any factor.

**Table:** Seroprevalence of bluetongue virus among red deer, by age and study area, Belgium, 2006–2008*

Characteristic	2006			2007			2008	
	No. positive/ no. tested (%)	95% CI		No. positive/ no. tested (%)	95% CI		No. positive/ no. tested (%)	95% CI
Age†								
Adults	4/221 (1.81)	0.05–3.57		142/216 (65.74)	59.41–72.07		111/185 (60.00)	52.94–67.06
Subadults	1/59 (1.69)	0.00–4.99		45/82 (54.88)	44.11–65.65		27/75 (36.00)	25.14–46.86
Juveniles	2/178 (1.12)	0.00–2.67		80/213 (37.56)	31.06–44.06		11/191 (5.76)	2.46–9.06
Area‡								
Eastern	ND	ND		81/161 (50.31)	42.59–58.03		43/123 (34.96)	26.53–43.39
Central	ND	ND		103/154 (66.88)	59.45–74.32		61/135 (45.19)	36.79–53.58
Southern	ND	ND		32/105 (30.48)	21.67–39.28		27/133 (20.30)	13.46–27.14

*CI, confidence interval; ND, not determined. †Adults, >2 y of age; subadults, 1–2 y of age; juveniles, <1 y of age. ‡Fourteen forest districts were distributed among 3 nonadjacent areas, 5 in the eastern area, 4 in the central area, and 5 in the southern area.

The 343 spleen samples (230 red deer and 113 roe deer) tested by RT-qPCR in 2006 and the 193 samples (roe deer) tested in 2007 were negative for BTV RNA. Conversely, ≈14% (33/237) of red deer β-actin–positive samples (237/331) were positive for BTV RNA (Technical Appendix). These 33 animals did not have gross lesions suggestive of bluetongue disease. Of 32 serum samples available, 26 were seropositive, 1 was doubtful, and 5 were seronegative, which suggested that these animals had been infected recently. Two pan-BTV RNA–positive spleen fragments, sampled at the end of hunting season in 2008, were reassessed by using a BTV-8 genotype–specific RT-qPCR (*4*); results were positive for all.

## Conclusions

Our study provides evidence that BTV-8 infects wild cervid populations in Belgium. For red deer, a few infections occurred in 2006 in the eastern part of Belgium, i.e., the area in which the ovine and bovine cases had been detected (*5*). In 2007, the infection spread west and southwest, and its seroprevalence increased. In 2008, distribution remained stable but overall seroprevalence decreased, mostly among juveniles. Distribution profiles of antibodies against BTV in 2007 and 2008 showed a bimodal profile. A large number of serum samples showed percentage negativity values between positive and negative values, which is indicative of ongoing virus transmission by vector midges during the hunting season. More animals were infected in eastern and central than in southern Wallonia, a finding that resembles the spatial distribution of the virus in livestock and might be correlated with lower density of cattle populations and cooler temperatures in hilly southern districts (*6*).

The proportion of seropositive animals increased with age, probably resulting more from prolonged exposure of adults to the vector, than to any resistant status of juveniles. Decreasing overall seroprevalence in 2008 might be caused by mandatory vaccination of domestic ruminants and spontaneously acquired herd immunity within the red deer population, which reduced virus prevalence among insect vector populations in 2008. Because seropositive subadults and adults sampled in 2008 could have been infected in 2007, seroprevalence among juveniles should more accurately reflect the level of exposure to infected insect vectors. If this explanation was true, the decrease in seroprevalence among juveniles in 2008 would confirm reduction of the virus insect reservoir.

Although red deer and roe deer samples were collected at the same locations and during the same hunting events, seroprevalence was lower among roe deer. Because 5 of 12 ELISA-positive and none of the ELISA-negative roe deer serum samples were positive by BTV-8 VN test and a similar between-species pattern was found by RT-qPCR, poor sensitivity of the ELISA in roe deer as the cause of between-species difference can be ruled out. Host-related and vector-related factors might account for this difference. Because red deer live in large groups and move more, they might be more exposed to insects/pathogens than are roe deer, which live in small groups in winter and have smaller home-range sizes and are seasonally territorial (*7*). Moreover, a recent follow-up of *Culicoides* spp. midge feeding patterns reported variations in host attractiveness, which could correlate with exhaled carbon dioxide and acetone (*8*), specific phenolic compounds emitted from urine (*9*) or hair fragrance (*10*).

BTV might be maintained in an as yet unknown reservoir host population with a long or relapsing viremia and in which clinical signs are absent. Because no excess illness or death occurred in 2007–2008, BTV-8 infection of wild cervids is probably benign enough to go unnoticed. Some spleen samples from dead red deer found during winter also showed positive results by RT-qPCR even if BTV was not the cause of death (A. Linden, unpub. data). Coupled with the high seroprevalence we report, the possibility that red deer are BTV reservoirs warrants further investigation.

## Supplementary Material

Technical Appendix Bluetongue Virus in Wild Deer, Belgium, 2005-2008
