# Supplementary material for: Bluetongue Virus in Wild Deer, Belgium, 2005–2008
Source: Emerg Infect Dis. 2010 May;16(5):833–6. doi: 10.3201/eid1605.091217 (PMC2953989; doi:10.3201/eid1605.091217)
Supplement: Technical Appendix — Bluetongue Virus in Wild Deer, Belgium, 2005-2008 [file 09-1217_Techapp-s1.pdf]

Bluetongue Virus in Wild Deer, Belgium, 2005–2008

Technical Appendix

Table 1. Distribution of hunter-killed wild cervids sampled, Wallonia, Belgium, 2005–2008*

Year	*Cervus elaphus*									*Capreolus capreolus*								
	Juveniles			Subadults		Adults		ND	Total	Juveniles			Subadults		ND			Total
	M	F	ND	M	F	M	F	–		M	F	ND	M	F	M	F	ND	
2005	49	32	1	19	18	32	45	–	196	6	12	–	14	27	–	–	–	59
2006	101	73	4	36	23	78	143	–	458	23	33	–	57	75	–	9	–	197
2007	111	101	1	43	39	86	130	1	512	40	45	2	75	119	4	7	3	295
2008	100	91	–	27	48	77	108	3	454	26	35	2	80	94	–	2	6	245
Total									1,620									796

*ND, not determined. Juveniles, <1 y of age; subadults, 1–2 y of age; adults, >1 y of age (*C. capreolus*) or 2 y of age (*C. elaphus*). Animals were sampled during hunting events during October 1 and December 31.

Table 2. Competitive ELISA and qRT-PCR results for BTV in red deer, Belgium, 2007*

ELISA % Negativity	qRT-PCR (spleen)					Total ELISA
	BTV RNA positive (14%)		BTV RNA doubtful (11%)	BTV RNA negative (75%)	β-actin negative (no RNA)	
	C_t 30–34	C_t 35–38	C_t 39–45	C_t >45		
Positive (180, 56%)						
0–5	0	4	0	0	0	4
6–15	5	4	6	17	14	46
16–25	2	4	8	19	14	47
26–35	3	3	3	16	15	40
36–45	0	0	2	13	4	19
46–55	0	1	2	5	3	11
56–65	0	0	1	8	4	13
Doubtful						
66–75	1	0	0	5	1	7
Negative (136, 42%)						
76–85	0	0	0	6	0	6
85–115	1	0	2	14	8	25
116–175	0	1	0	43	19	63
>175	1	2	3	24	12	42
Blood not available	0	1	0	7	0	8
Total qRT-PCR	13	20	27	177	94	
	237				94	331

*Data are animal samples from the hunting season. qRT-PCR, quantitative reverse transcription–PCR; BTV, bluetongue virus; C_t, cycle threshold.
